# ParetoEnsembles.jl: A Julia Package for Multiobjective Parameter Estimation Using Pareto Optimal Ensemble Techniques

**Published:** 2026-03-31

**Authors:** Jeffrey D. Varner

**Affiliations:** 1 Robert Frederick Smith School of Chemical and Biomolecular Engineering, Cornell University, Ithaca, NY 14853, USA

**Keywords:** multiobjective optimization, Pareto front, simulated annealing, ensemble methods, parameter estimation, uncertainty characterization

## Abstract

Mathematical models of natural and man-made systems often have many adjustable parameters that must be estimated from multiple, potentially conflicting datasets. Rather than reporting a single best-fit parameter vector, it is often more informative to generate an *ensemble* of parameter sets that collectively map out the trade-offs among competing objectives. This paper presents ParetoEnsembles.jl, an open-source Julia package that generates such ensembles using Pareto Optimal Ensemble Techniques (POETs), a simulated-annealing-based algorithm that requires no gradient information. The implementation corrects the original dominance relation from weak to strict Pareto dominance, reduces the per-iteration ranking cost from $O\left(n^{2}m\right)$ to O(nm) through an incremental update scheme, and adds multi-chain parallel execution for improved front coverage. We demonstrate the package on a cell-free gene expression model fitted to experimental data and a blood coagulation cascade model with ten estimated rate constants and three objectives. A controlled synthetic-data study reveals parameter identifiability structure, with individual rate constants off by several-fold yet model predictions accurate to 6–7%. A five-replicate coverage analysis confirms that timing features are reliably covered while peak amplitude is systematically overconfident. Validation against published experimental thrombin generation data demonstrates that the ensemble predicts held-out conditions to within 1–10% despite inherent model approximation error. By making ensemble generation lightweight and accessible, ParetoEnsembles.jl aims to lower the barrier to routine uncertainty characterization in mechanistic modeling.

## 1 Introduction

Mathematical models of biological and engineering systems typically contain many adjustable parameters that must be estimated from experimental data. When multiple datasets measure different aspects of the same system (for example, mRNA and protein concentrations in a gene expression circuit, or yield and selectivity in a reactor), calibrating parameters to one dataset often degrades the fit to another. This gives rise to a multiobjective optimization (MOO) problem [1–3]. Compounding this difficulty, many models exhibit “sloppy” parameter sensitivities: broad, flat valleys in objective space where large regions of parameter space produce nearly equivalent fits [4]. In such settings, a single best-fit parameter vector gives no indication of how tightly the data actually constrain the parameters; instead, one needs an *ensemble* of parameter sets that collectively map out the trade-offs among competing objectives. The boundary of this ensemble in objective space is the Pareto front, the set of solutions for which no objective can be improved without degrading at least one other, and the full cloud of near-optimal solutions surrounding it characterizes the range of model behaviors consistent with the available data.

Several algorithm families have been developed to approximate Pareto fronts. Population-based evolutionary methods maintain a generation of candidate solutions that are selected, recombined, and mutated toward the front. Prominent examples include the Non-dominated Sorting Genetic Algorithm II (NSGA-II) [5], its many-objective successor NSGA-III [6], and the Multiobjective Evolutionary Algorithm Based on Decomposition (MOEA/D), which decomposes the MOO problem into a set of scalar subproblems solved cooperatively [7]. An alternative trajectory-based strategy is multiobjective simulated annealing (SA), which extends the classical SA framework [8] to multiple objectives by using Pareto dominance to guide acceptance decisions; early formulations include the Pareto SA method of Czyżak and Jaszkiewicz [9] and the Archived Multiobjective Simulated Annealing (AMOSA) algorithm of Bandyopadhyay et al. [10]. Pareto Optimal Ensemble Techniques (POETs), introduced by Song et al. [11] and later implemented in the Julia programming language by Bassen and colleagues [12], belongs to this SA-based family. The POETs algorithm ranks the current archive by counting how many other solutions dominate each candidate, then uses the rank as the energy in a Metropolis-type acceptance criterion. Mature software implementations of these and related algorithms are available in several languages, including pymoo in Python [13] and Metaheuristics.jl in Julia [14], both of which focus primarily on population-based evolutionary strategies. A complementary approach to characterizing parametric uncertainty is Bayesian inference, where Markov chain Monte Carlo (MCMC) or sequential Monte Carlo (SMC) methods sample from the posterior distribution over parameters given the data. Widely used implementations include Stan [15], Turing.jl [16], and the ensemble MCMC sampler emcee [17]. While Bayesian methods provide a formal probabilistic interpretation of parameter uncertainty, they require specification of prior distributions and a scalar likelihood, scale poorly with the number of objectives, and can be expensive for stiff ODE models. On the other hand, multiobjective ensemble methods such as POETs offer a lightweight alternative that naturally handles multiple competing objectives and returns a cloud of near-optimal solutions without requiring probabilistic modeling assumptions.

In this study, we present ParetoEnsembles.jl, a substantially rewritten Julia implementation of the POETs algorithm [11, 12]. The package takes a user-defined model and a set of objective functions, each measuring how well a candidate parameter vector reproduces a particular dataset, and returns a collection of parameter sets spanning the trade-off surface, along with their objective values and dominance ranks. Several algorithmic corrections and improvements have been made relative to the original implementation: the dominance check has been corrected from weak to strict Pareto dominance, the per-iteration ranking cost has been reduced from $O\left(n^{2}m\right)$ to O(nm) through an incremental update scheme (where n is the archive size and m is the number of objectives), and multi-chain parallel execution has been added to improve front coverage. We demonstrate the package on two biological systems of increasing complexity (Section 3), validate it against standard benchmarks and NSGA-II, and assess hyperparameter sensitivity (Sections S4 to S6). By making ensemble generation lightweight and accessible, the package lowers the barrier to routine uncertainty characterization in mechanistic modeling.

## 2 Methods

### 2.1 Pareto dominance and ranking

Consider an archive of n solutions evaluated on m objectives, where lower values are preferred. Solution ${\text{x}}_{j}$ *strictly dominates* solution ${\text{x}}_{i}$, written ${\text{x}}_{j}≺{\text{x}}_{i}$, if and only if $f_{k}\left({\text{x}}_{j}\right)\leq f_{k}\left({\text{x}}_{i}\right)$ for all k∈{1,…,m} and there exists at least one objective k for which $f_{k}\left({\text{x}}_{j}\right)<f_{k}\left({\text{x}}_{i}\right)$ (Equation (1)). The *Pareto rank* of solution ${\text{x}}_{i}$ is the number of archive members that strictly dominate it (Equation (2)), so that solutions with rank zero lie on the Pareto front and identical solutions, which do not dominate each other under strict dominance, share the same rank. The original POETs implementation [11, 12] used *weak* dominance, checking only that $f_{k}\left({\text{x}}_{j}\right)\leq f_{k}\left({\text{x}}_{i}\right)$ for all k without requiring strict inequality in at least one objective; under weak dominance, identical solutions count as dominating each other, which inflates their ranks and causes excessive pruning of good solutions from the archive. The current implementation enforces strict Pareto dominance. The pairwise test iterates over objectives with an early-exit condition that terminates as soon as any objective of j is found to be worse than i, avoiding unnecessary comparisons (pseudocode in Algorithm S2).


(1) $$f_{k}\left({\text{x}}_{j}\right)\leq f_{k}\left({\text{x}}_{i}\right)\;\forall k\in \{1,\ldots ,m\}\;\text{and}\;\exists k:f_{k}\left({\text{x}}_{j}\right)<f_{k}\left({\text{x}}_{i}\right).$$

(2) $$R\left({\text{x}}_{i}\right)=\left|\left\{j\neq i|{\text{x}}_{j}≺{\text{x}}_{i}\right\}\right|.$$

### 2.2 Incremental ranking

A central computational concern is the cost of ranking. When a single candidate solution ${\text{x}}_{\text{new}}$ is appended to an archive of n existing solutions with known ranks R, the naïve approach recomputes all pairwise dominance checks at $O\left(n^{2}m\right)$ cost, where m is the number of objectives. Because only one solution changed, however, we can instead perform a single O(nm) pass over the existing archive: for each existing solution ${\text{x}}_{i}$, we test dominance in both directions, incrementing $R_{i}$ if the new solution dominates ${\text{x}}_{i}$ and incrementing the new solution’s rank if ${\text{x}}_{i}$ dominates it. This incremental procedure reduces the per-iteration cost from $O\left(n^{2}m\right)$ to O(nm), with a full $O\left(n^{\prime 2}m\right)$ re-rank performed only after removing high-rank solutions from the archive, where $n^{\prime }$ is the reduced archive size (pseudocode in Algorithm S3).

### 2.3 Pareto simulated annealing

The complete Pareto simulated annealing procedure combines the dominance check and incremental ranking into a nested loop over temperature levels and candidate evaluations. The algorithm is seeded with an initial parameter vector ${\text{x}}_{0}$, an initial temperature T=1, and an archive containing the single evaluated solution $\left({\text{x}}_{0},\text{f}\left({\text{x}}_{0}\right)\right)$. At each temperature level, $N_{\text{iter}}$ candidate solutions are generated by perturbing the current best parameter vector through a user-supplied neighbor function, and the rank array is updated incrementally at O(nm) cost per candidate. The acceptance probability, computed from the rank array and the current temperature, determines whether the candidate is retained; upon acceptance, solutions whose rank meets or exceeds the cutoff $R_{\text{cutoff}}$ are removed and a full re-rank is performed on the remaining set. If the archive still exceeds the maximum size $n_{\max}$, only the $n_{\max}$ lowest-rank solutions are retained. Upon rejection, the candidate is immediately removed and the rank array is restored from a saved snapshot, a strategy we refer to as *pop-on-reject*. In the original implementation, rejected candidates remained in the archive, causing unbounded growth of n between accepted moves and progressively more expensive ranking operations; the pop-on-reject strategy eliminates this problem. To improve front coverage, multiple independent SA chains can be run from different starting points and their archives merged, with a final re-rank performed on the combined set. This multi-chain strategy provides near-linear parallel speedup and ensures diverse exploration of the trade-off surface (pseudocode for the main loop and multi-chain wrapper in Algorithms S1 and S4; software design details and the public API in Section S2).

## 3 Results

We first validated ParetoEnsembles.jl on two standard multiobjective benchmarks, the constrained Binh–Korn problem [18] and the unconstrained Fonseca–Fleming problem [19], recovering the known Pareto fronts in both cases (Section S4). A head-to-head comparison with NSGA-II [5] at matched evaluation budgets (~110,000) showed that ParetoEnsembles.jl achieved comparable hypervolume (the volume of objective space covered by the front, where larger is better), 5–7× lower inverted generational distance (the average distance from the true front to the computed front, where lower indicates a closer approximation), and 10–30× denser fronts with thousands of retained solutions compared with ~200 for NSGA-II (Section S5). A hyperparameter sensitivity analysis confirmed robustness to the choice of rank cutoff, cooling rate, and iterations per temperature (Section S6). Thus, ParetoEnsembles.jl produced denser and more complete approximations of the trade-off surface, but at the cost of longer wall-clock times (6–150× slower than NSGA-II, depending on problem size) due to the inherently sequential nature of simulated annealing chains. Next, we focus on two biological applications that illustrate the practical value of ensemble-based parameter estimation.

### 3.1 Cell-free gene expression

**Figure 1:**
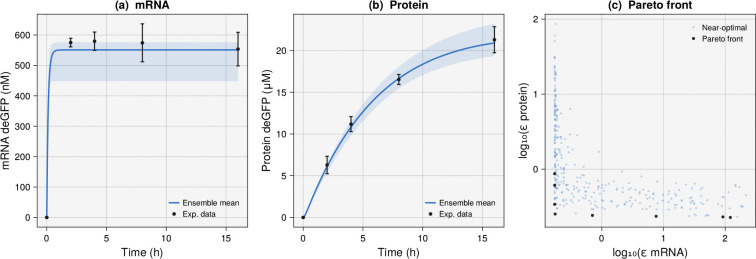
Ensemble estimation for a cell-free gene expression circuit ($\sigma_{70}\to \text{P}70\to \text{deGFP}$) fitted to experimental data from Adhikari et al. [21]. (a) mRNA and (b) protein concentration versus time; the blue dashed line is the ensemble mean, the shaded region is the 95% confidence interval, and black points are experimental data with error bars. (c) Pareto front showing the trade-off between mRNA and protein fitting error.

As a first biological application, we considered the estimation of kinetic parameters for a cell-free gene expression circuit. The circuit describes the production of deGFP, a highly translatable variant of enhanced green fluorescent protein developed by Shin and Noireaux [20], driven by the endogenous sigma factor $\sigma_{70}$ via the P70 promoter. We followed the effective biophysical framework of Adhikari et al. [21] (Figure 1). The system is governed by two ordinary differential equations for mRNA (m) and protein (p), where $\dot{m}=\alpha u\left(\sigma_{70}\right)-\delta_{m}m$ and $\dot{p}=\kappa mw(t)-\delta_{p}p$, with $u\left(\sigma_{70}\right)=\sigma_{70}^{n}/\left(K^{n}+\sigma_{70}^{n}\right)$ representing a Hill-type promoter activity function and $w(t)=\exp\left(-\ln2\cdot t/\tau_{1/2}\right)$ capturing the experimentally observed decay of translational capacity over the course of the cell-free reaction. Five parameters were treated as unknown (the maximum transcription rate α, the effective translation rate constant κ, the mRNA degradation rate $\delta_{m}$, the promoter dissociation constant K, and the translation capacity half-life $\tau_{1/2}$) while the remaining parameters ($\sigma_{70}=35\;\text{nM},n=1.5,\delta_{p}=0.005\;{\text{h}}^{-1}$) were fixed at values taken from the literature.

We fitted the model to experimental data from Adhikari et al. [21], consisting of mRNA and deGFP protein concentrations measured at five time points (0, 2, 4, 8, and 16 h) in triplicate, with reported means and standard deviations. Two objectives were defined as standard-deviation-weighted sums of squared errors: $\varepsilon_{\text{mRNA}}=\sum_{i}{\left[\left(m_{i}^{\text{sim}}-m_{i}^{\text{obs}}\right)/\sigma_{i}^{m}\right]}^{2}$ and $\varepsilon_{\text{protein}}=\sum_{i}{\left[\left(p_{i}^{\text{sim}}-p_{i}^{\text{obs}}\right)/\sigma_{i}^{p}\right]}^{2}$, and we ran ten parallel chains with a rank cutoff of $R_{\text{cutoff}}=8$, $N_{\text{iter}}=50$ candidate solutions per temperature, and a cooling rate of α=0.90, starting from random initial parameter vectors drawn uniformly within the parameter bounds specified in the original study [21]. The resulting ensemble captured the dynamics of both mRNA and protein expression (Figure 1a,b): the 95% confidence interval of the ensemble simulations bracketed the experimental data at all time points, and the ensemble mean tracked the observed trajectories. The Pareto front revealed a clear trade-off between fitting the mRNA and protein time courses (Figure 1c), with parameter sets that minimized $\varepsilon_{\text{mRNA}}$ tending to have larger $\varepsilon_{\text{protein}}$ and vice versa. This trade-off arises naturally because the mRNA degradation rate and translation capacity half-life have opposing effects on the two species. The ensemble provided a family of plausible parameter sets that traded off between these competing objectives, rather than a single point estimate that favors one objective over the other. Even for this small system, the ensemble revealed trade-off structure among the estimated parameters; we next ask whether the approach scales to a substantially larger and more clinically relevant model.

### 3.2 Blood coagulation cascade

**Figure 2:**
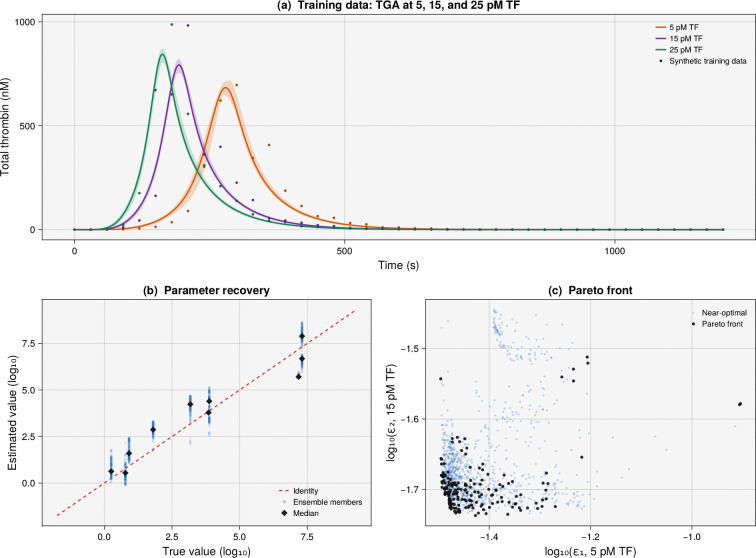
Training results for the Hockin–Mann blood coagulation model (34 species, 10 estimated rate constants, 3 data-driven objectives, no regularization). (a) Total thrombin concentration versus time at 5 pM (amber), 15 pM (purple), and 25 pM (teal) tissue factor; shaded regions are the ensemble 95% CI, dashed lines are the ensemble mean, and points are noisy synthetic data (15% CV). (b) Parameter recovery in log-space; the dashed red line is the identity and diamonds show the median ensemble estimate; scatter around each parameter reveals the degree of identifiability from thrombin data alone. (c) Pareto front projection ($\varepsilon_{1}$ vs. $\varepsilon_{2}$, colored by $\varepsilon_{3}$), with rank-zero solutions in black.

To demonstrate the package on a larger-scale system, we considered the Hockin–Mann model of tissue-factor-initiated blood coagulation [22] (Figure 2). This mechanistic ODE model describes 34 chemical species interacting through 27 reactions governed by 42 rate constants, capturing the initiation, amplification, and inhibition phases of thrombin generation. All rate constants are available in the literature and were used as ground truth. We selected ten key catalytic and inhibition rate constants for estimation spanning the extrinsic factor Xase $\left(k_{\text{cat}}\right)$, intrinsic factor Xase $\left(k_{\text{cat}}\right)$, prothrombinase $\left(k_{\text{cat}}\right)$, and antithrombin III inhibition pathways. Because these rate constants span six orders of magnitude (from ~1 to ~ 10^7^), we worked in log-space with bounds of ±1.5 orders of magnitude around the literature values. We generated noisy synthetic thrombin generation assay (TGA) data by simulating the model with the true rate constants at three tissue factor concentrations (5, 15, and 25 pM) and adding multiplicative Gaussian noise $\left(x^{\prime }=x\cdot (1+\epsilon ),\epsilon \sim 𝒩\left(0,0.15^{2}\right)\right)$ to the total thrombin trajectory at each condition. Three objectives were defined, the normalized sum-of-squared error of the thrombin trajectory at each of the three TF concentrations, with no regularization terms; the parameter recovery reflects only what the training data can constrain rather than being aided by a penalty that pulls estimates toward the known truth.

We ran eight parallel chains with a rank cutoff of $R_{\text{cutoff}}=10$, $N_{\text{iter}}=40$ candidate solutions per temperature, and a cooling rate of *α* = 0.92. The resulting ensemble of 222 parameter sets (rank ≤ 1) produced thrombin trajectories that closely matched the noisy training data at all three TF concentrations (Figure 2a), with 95% confidence intervals that bracketed the data throughout the time course. However, parameter recovery was mixed, revealing the practical identifiability structure of the system (Figure 2b). Some rate constants were well recovered (the IIa+ATIII inhibition rate to within 2% and the Factor Va activation rate to within 5%), while others were poorly constrained by thrombin data alone: the intrinsic factor Xase $k_{\text{cat}}$ was off by a factor of five and the direct Xa-mediated thrombin generation rate $\left(k_{\text{Xa}\to \text{IIa}}\right)$ was overestimated by more than twofold. The three-dimensional Pareto front (Figure 2c) revealed the expected trade-offs among fitting the three TF conditions, demonstrating that ParetoEnsembles.jl can handle systems of moderate complexity with tens of species, ten or more estimated parameters, and three competing objectives. Several parameters were poorly recovered, yet the model still fit the training data well: this is consistent with compensatory adjustments among rate constants producing nearly identical thrombin trajectories from very different parameter vectors. This motivates the ensemble-based downstream study described next.

### 3.3 Ensemble-based prediction and identifiability analysis

**Figure 3:**
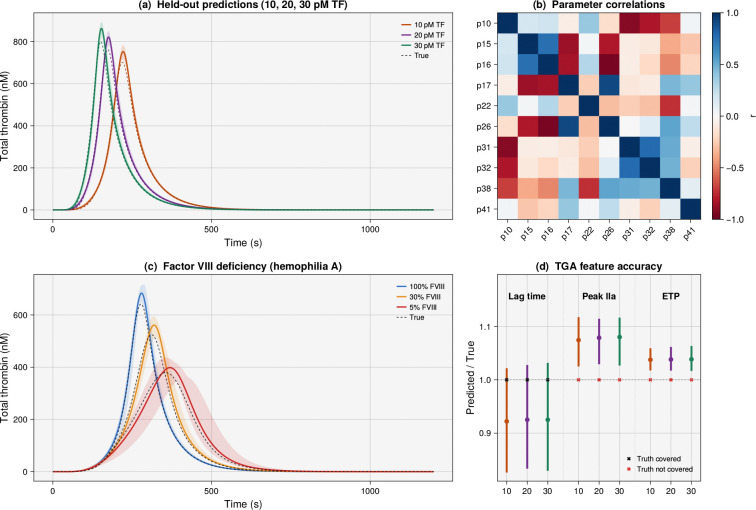
Ensemble-based uncertainty characterization for the coagulation model. (a) Heldout thrombin predictions at 10, 20, and 30 pM TF (not used during training); solid lines are true trajectories, dashed lines are ensemble means, and shaded regions are 95% CIs. The ensemble captures the overall shape but shows a systematic positive bias at peak thrombin. (b) Pairwise parameter correlation heatmap revealing extensive compensatory structure; strong negative correlations (e.g., extrinsic Xase $k_{\text{cat}}$ vs. prothrombinase $k_{\text{cat}}$, r=−0.81; Xa→IIa vs. IIa→Va, r=−0.93) explain why individual parameters can be poorly recovered while model predictions remain accurate. (c) Patient-specific predictions for Factor VIII deficiency (hemophilia A) at 100%, 30%, and 5% of nominal FVIII levels; ensemble prediction bands capture the dose-dependent reduction in peak thrombin, and true trajectories (black dashed) fall within all three bands. (d) TGA feature accuracy at held-out conditions: ensemble-predicted lag time, peak thrombin, and ETP normalized to true values, with 95% CIs; black crosses indicate the true value is covered by the interval, red crosses indicate it is not.

A key advantage of generating parameter ensembles rather than single point estimates is the ability to propagate uncertainty forward through the model to obtain prediction intervals whose width reflects what the training data can and cannot constrain (Figure 3). To test this, we used the coagulation ensemble, which was trained only on TGA curves at 5, 15, and 25 pM TF, to predict the full thrombin time course at three held-out tissue factor concentrations (10, 20, and 30 pM) that were never used during optimization. The ensemble mean tracked the true trajectories at all three conditions, predicting peak thrombin to within 6–7% of the true values, and the 95% prediction intervals captured the overall shape of the thrombin generation curve (Figure 3a). However, the prediction bands did not fully bracket the true trajectories at the peak, revealing a systematic positive bias in peak amplitude that the ensemble’s uncertainty did not account for. The ensemble made this systematic bias visible by providing prediction intervals whose width varied across outputs and conditions. To quantify this further, we extracted three clinically relevant TGA features (lag time, peak thrombin concentration, and endogenous thrombin potential (ETP)) from both the ensemble predictions and the true trajectories at each held-out condition (Figure 3d). Lag time was well predicted with appropriately wide 95% intervals that covered the true value at all three concentrations, while peak thrombin and ETP showed a consistent 4–7% overprediction with intervals too tight to achieve coverage, indicating that the ensemble is overconfident about thrombin amplitude even as it correctly captures the timing of the coagulation response.

Pairwise correlations in the ensemble revealed the model’s identifiability structure (Figure 3b). The strongest negative correlation was between the extrinsic factor Xase $k_{\text{cat}}$ and the prothrombinase $k_{\text{cat}}(r=-0.81)$, indicating compensatory behavior: when the initiation-phase enzyme is faster, the amplification-phase enzyme must be slower to produce the same thrombin trajectory. Similar compensatory couplings appeared between the direct Xa-mediated thrombin generation rate and Factor Va activation (r=−0.93), reflecting competition between direct and prothrombinase-mediated pathways, and between the TF=VIIa→IX catalytic rate and the mIIa→IIa conversion rate (r=−0.73), linking initiation and propagation. These relationships explain why individual parameters can be off by several-fold while model predictions remain accurate to within a few percent: correlated shifts in one rate constant are offset by compensatory changes in others, so the ensemble spans a region of parameter space that produces similar trajectories despite wide variation in individual parameters.

Finally, we used the ensemble to simulate a clinically relevant perturbation: Factor VIII deficiency (hemophilia A) at varying severity levels. By reducing the initial Factor VIII concentration to 30% (mild hemophilia) and 5% (severe hemophilia) of the nominal plasma level while keeping all estimated rate constants at their ensemble values, we generated virtual-patient predictions of thrombin generation that captured the expected dose-dependent reduction in peak thrombin and associated uncertainty (Figure 3c). Peak thrombin decreased from 684 ± 22 nM in normal plasma to 563 ± 22 nM for mild hemophilia and 401 ± 21 nM for severe hemophilia, with the true trajectories falling within the ensemble prediction bands for all three conditions. The prediction intervals widened as Factor VIII was reduced, consistent with the expectation that the model becomes less constrained as the system is driven further from the conditions used for training. The ensemble correctly bracketed the true trajectories for these out-of-distribution perturbations despite poor recovery of several individual rate constants, suggesting that ensemble-based uncertainty characterization may be useful in translational settings where model predictions inform clinical decisions. Taken together, these analyses showed that the ensemble revealed identifiability structure, propagated uncertainty to held-out conditions, and supported clinically relevant perturbation studies; but all relied on synthetic data where the model was correct by construction. To test robustness to model error, we repeated the coagulation study with six fixed rate constants perturbed by ±30%; the ensemble still predicted held-out peak thrombin to within 4–6%, though trajectory-level coverage degraded to 31–48% (Section S7). We next test whether these findings hold when the model is only an approximation of reality.

### 3.4 Validation against experimental data

**Figure 4:**
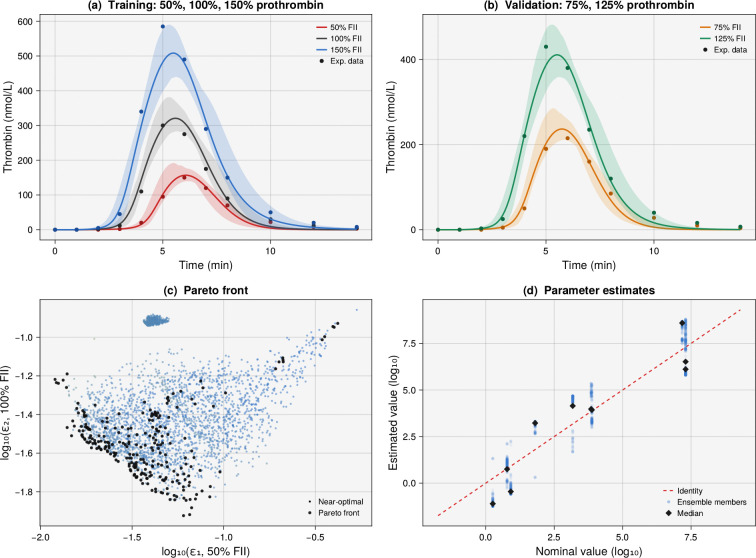
Ensemble estimation fitted to experimental thrombin generation data from Butenas et al. [23]. Prothrombin was varied from 50% to 150% of its mean plasma concentration in a reconstituted synthetic plasma system initiated by 5 pmol/L TF–VIIa. (a) Training fits at 50% (red), 100% (gray), and 150% (blue) prothrombin; the ensemble captures the experimental data at low and normal prothrombin levels but underestimates the peak at 150%, revealing a model–data tension. (b) Held-out validation at 75% (amber) and 125% (teal) prothrombin; the ensemble predicts peak thrombin to within 1–10% at conditions never used during training. (c) Pareto front projection showing trade-offs among the three training objectives. (d) Parameter estimates versus nominal literature values; deviations reflect compensatory adjustments to fit experimental data with an approximate model.

The coagulation analyses above used synthetic data where the ground truth was known, enabling systematic assessment of parameter recovery and coverage. However, these tests left open the question of whether the ensemble approach works when the model is only an approximation of reality. To address this, we fitted the Hockin–Mann model to published experimental data from Butenas et al. [23], who measured thrombin generation in a reconstituted synthetic plasma system at five prothrombin concentrations ranging from 50% to 150% of the mean plasma value, with all other coagulation factors at their nominal concentrations; time-course data were digitized from Figure 3 of [23], with peak values calibrated against Table 2. This system provided a suitable test case because the initial conditions were well characterized (Table 1 of [23]), the protein C pathway was absent (matching the Hockin–Mann model), and the prothrombin range spans a clinically relevant source of interindividual variability (Figure 4).

We defined three training objectives as the normalized sum of squared errors (SSE) between simulated and experimental thrombin time courses at 50%, 100%, and 150% of normal prothrombin. We held out the 75% and 125% conditions for validation. Eight parallel chains were run with the same hyperparameters as the synthetic study, yielding an ensemble of 390 parameter sets (rank ≤ 1). The ensemble captured the experimental thrombin profiles at the training conditions (Figure 4a), with peak thrombin predicted to within 0.1–10% across the three prothrombin levels; the residual discrepancies reflected genuine model–data tension in which the Hockin–Mann model’s sensitivity to prothrombin concentration did not match the experimental system, a mismatch that was visible in the trade-off structure of the Pareto front (Figure 4c). At the held-out validation conditions, the ensemble predicted peak thrombin at 75% and 125% prothrombin to within 1–10% of the experimental values (Figure 4b), with the ensemble mean tracking the data closely and the 95% prediction intervals bracketing the experimental time points at both conditions. The estimated rate constants deviated from their nominal literature values (Figure 4d), as expected when fitting real data where the model structure is only an approximation of the underlying biochemistry; these deviations reflected compensatory adjustments in the estimated parameters to offset differences between the model and the experimental system. The spread of the ensemble around each parameter indicated how tightly the data constrained that rate constant. These results confirmed that the ensemble approach generalizes beyond the synthetic setting: even when the model is an imperfect representation of the underlying system, the method produced accurate held-out predictions with informative uncertainty bands.

## 4 Discussion

We presented ParetoEnsembles.jl, a lightweight Julia implementation of Pareto Optimal Ensemble Techniques (POETs) for multiobjective optimization that addressed several limitations of the original implementation [11, 12]. The current version corrected the dominance relation from weak to strict, reduced the per-iteration ranking cost from $O\left(n^{2}m\right)$ to O(nm) through incremental updating, bounded archive growth through the pop-on-reject strategy and a hard size cap, added both threaded ranking and multi-chain parallel execution, and provided built-in hypervolume computation and convergence diagnostics. The callback-based design decoupled the optimization algorithm from the problem definition, allowing users to apply ParetoEnsembles.jl to any domain where objective functions can be evaluated pointwise. On standard benchmark problems, ParetoEnsembles.jl produced fronts with hypervolume and inverted generational distance competitive with NSGA-II at matched evaluation budgets, while retaining ensembles that were 10–30× denser than those returned by NSGA-II (Section S5). This density mattered in practice: meaningful computation of prediction intervals, parameter correlations, and coverage statistics from an ensemble required hundreds to thousands of members that fill the near-optimal region of parameter space, not just the 100–200 non-dominated solutions typical of a population-based method. While NSGA-II was considerably faster in wall time, reflecting the inherently sequential nature of SA chains versus the population-parallel evaluation of evolutionary methods, ParetoEnsembles.jl targeted problems where the goal was to characterize the *cloud* of near-optimal parameter sets, not just the front itself. Further, the simplicity of the interface lowered the barrier to generating parameter ensembles as part of a modeling workflow.

The biological applications presented here highlight why ensemble-based parameter estimation differs from conventional single-point optimization. The cell-free gene expression example demonstrated that even a small model with five parameters and two objectives revealed meaningful trade-offs when fitted to real experimental data, and the coagulation cascade example showed that the approach scaled to larger systems with ten parameters spanning six orders of magnitude and three competing objectives. A central finding of the coagulation study was that accurate model predictions do not require accurate parameter recovery. The ensemble predicted thrombin generation at held-out TF concentrations to within 6–7% of the true peak even though individual rate constants were off by as much as fivefold, because pairwise parameter correlations revealed extensive compensatory structure. The pairwise parameter correlations revealed that many different parameter combinations produced nearly identical thrombin trajectories. The held-out validation also exposed a limitation: while the ensemble correctly predicted the timing of thrombin generation (lag time covered at all conditions), it showed a systematic positive bias in peak amplitude and ETP with confidence intervals too tight to achieve nominal coverage, suggesting that additional data types would be needed to fully constrain the amplitude of the thrombin burst. This controlled experiment validated the algorithm’s ability to explore the trade-off surface and provided a ground-truth benchmark for assessing coverage and identifiability, but it did not test robustness to model misspecification. To address this, we repeated the study with six fixed rate constants perturbed by ±30% so that the fitting model was misspecified (Section S7); the ensemble still predicted held-out peak thrombin to within 4–6%, though trajectory-level coverage degraded to 31–48%, confirming that the approach is robust to moderate structural error while revealing that parametric uncertainty alone cannot account for model inadequacy. The hemophilia A simulations demonstrated that the ensemble can propagate parametric uncertainty through clinically relevant perturbations, with prediction intervals that widen as the system is driven further from training conditions and that correctly bracket the true trajectories even when the underlying parameters are individually poorly identified. These results provide a template for how ParetoEnsembles.jl can be used in practice. The hyperparameter sensitivity analysis (Section S6) further showed that the algorithm was robust to the choice of rank cutoff, cooling rate, and iterations per temperature, so that users can adopt the default settings without extensive tuning, while the convergence trace feature provided a built-in diagnostic for monitoring annealing progress.

The ensemble produced by ParetoEnsembles.jl is not a Bayesian posterior distribution. The ensemble is a collection of parameter vectors that are near-optimal with respect to multiple objectives, selected by simulated annealing with rank-based acceptance; it does not correspond to samples from a probability distribution conditioned on the data, and the 95% intervals reported throughout this paper are empirical quantiles of the ensemble predictions, not Bayesian credible intervals. The ensemble makes no claim about the relative probability of different parameter vectors, only that they produce comparably good fits, and its coverage properties depend on how thoroughly the simulated annealing chains explore the near-optimal region rather than on convergence to a stationary distribution.Bayesian approaches such as MCMC [15, 16] or ensemble MCMC [17] provide a principled probabilistic framework for parameter uncertainty, but they require specification of prior distributions and a scalar likelihood function, which is not naturally defined when multiple incommensurable objectives must be balanced simultaneously; they also face well-known challenges with multimodal posteriors and stiff ODE likelihoods that can make convergence slow or unreliable. The Pareto ensemble approach sidesteps these difficulties at the cost of probabilistic interpretability, and users who require calibrated posterior credible intervals should consider Bayesian methods as a complementary analysis. A promising direction for bridging the two frameworks is the Gibbs posterior, where the loss-based objectives used here replace the log-likelihood in a generalized Bayesian update; importance reweighting of the ensemble members against such a posterior could recover calibrated credible intervals while reusing the expensive model evaluations already performed during optimization. To verify that the coverage patterns are systematic rather than artifacts of a single noise realization, we repeated the synthetic coagulation study five times with independent noise seeds and computed feature-level coverage rates across replicates. The results confirmed a clear and reproducible pattern: lag time and time-to-peak achieved 100% coverage (5/5 replicates, all held-out conditions), while peak thrombin was covered in only 40% of replicates and ETP in 60%, with trajectory-level coverage averaging 87–90% across conditions. The mean held-out peak error was 7.1 ± 5.0% (range 0.9–17.9%), confirming that the single-replicate results reported above are representative. This systematic pattern, in which timing is well-calibrated but amplitude is overconfident, likely reflects the concentration of the ensemble along the compensatory structure identified by the correlation analysis: the simulated annealing chains efficiently explore directions in parameter space along which objective values change (i.e., along the Pareto front), but they may underexplore directions orthogonal to the front where objective values are nearly constant yet model predictions differ, leading to prediction intervals that are well-calibrated for some outputs but overconfident for others. We also verified that the downstream predictions are insensitive to the choice of which archive members are included in the ensemble: sweeping the rank cutoff from 0 (Pareto front only) through rank ≤ 5 changed the mean held-out peak error by less than 0.2 percentage points, confirming that the near-optimal solutions surrounding the front carry essentially the same predictive information as the front itself.

Several features were deliberately omitted from the current scope. The package does not implement gradient-based refinement, surrogate modeling, or adaptive cooling schedules, nor does it provide built-in visualization or benchmark problem libraries. Future directions include integration with Julia’s automatic differentiation ecosystem (e.g., ForwardDiff.jl or Enzyme.jl) to enable hybrid gradient-free/gradient-based strategies for problems where gradients are available for some but not all objectives, adaptive archive management using hypervolume indicators [24] in place of simple rank-based pruning for more principled control over the distribution of retained solutions, and a formal connection to Bayesian inference through the Gibbs posterior framework discussed above. Despite these limitations, the current version provides a minimal and accessible tool for generating Pareto-optimal parameter ensembles, and we anticipate that it will be useful for researchers in systems biology and beyond who need to characterize uncertainty in mechanistic models.

## 5 Conclusions

In this study we presented ParetoEnsembles.jl, a Julia package for generating Pareto-optimal parameter ensembles using simulated annealing with incremental dominance ranking. The package corrects a longstanding weak dominance issue in the original POETs implementation, reduces the per-iteration ranking cost from quadratic to linear in the archive size, and adds multi-chain parallel execution that provides improved front coverage from diverse starting points. The package was applied to two biological systems of increasing complexity: a cell-free gene expression circuit fitted to experimental mRNA and protein time-course data, and the Hockin–Mann blood coagulation cascade model with 34 species and ten estimated rate constants spanning six orders of magnitude. A controlled synthetic-data study revealed that accurate model predictions do not require accurate parameter recovery, as the ensemble predicted held-out thrombin generation to within 6–7% despite individual parameters being off by several-fold. Identifiability analysis, patient-specific hemophilia A simulations, and a model misspecification study (Section S7) collectively demonstrated that ensemble-based uncertainty characterization reflects what the data can and cannot constrain. Validation against published experimental thrombin generation data from Butenas et al. [23] confirmed that the approach generalizes beyond the synthetic setting: the ensemble trained on three prothrombin levels predicted held-out conditions to within 1–10%, even though the model is only an approximation of the underlying experimental system. ParetoEnsembles.jl is open source, registered in the Julia General registry, and can be installed with a single command; we anticipate that its minimal interface and dependency-free design will make it a useful tool for researchers who need to generate parameter ensembles for mechanistic models in systems biology and beyond.

## Supplementary Material

Supplement 1

## Data Availability

ParetoEnsembles.jl is open source under the MIT license. The source code is available at https://github.com/varnerlab/ParetoEnsembles.jl, and documentation is hosted at https://varnerlab.github.io/ParetoEnsembles.jl/dev/. The package is registered in the Julia General registry and can be installed via Pkg.add(“ParetoEnsembles”). Example code reproducing all results in this paper is included in the paper/code directory of the source repository.
