# Supplementary material for: ParetoEnsembles.jl: A Julia Package for Multiobjective Parameter Estimation Using Pareto Optimal Ensemble Techniques
Source: ArXiv. 2026 Mar 31:arXiv:2603.29986v1. Preprint. [Version 1] (PMC13060479)
Supplement: Supplement 1 [file NIHPP2603.29986v1-supplement-1.pdf]

Supplementary Material

S1 Algorithm pseudocode

This section provides complete pseudocode for the four algorithmic components of ParetoEnsembles.jl. We begin with the main simulated annealing loop, which orchestrates the optimization, and then present the subroutines it relies on: the strict dominance test that underlies all ranking decisions, the incremental rank update that keeps per-iteration cost linear, and the multi-chain wrapper that parallelizes the search.

Main algorithm. The core of ParetoEnsembles.jl is a Pareto simulated annealing loop (Algorithm S1) that maintains an archive of evaluated solutions and their Pareto ranks. At each temperature level, candidates are generated by perturbing the current best parameter vector, and the archive is updated incrementally via RANKINSERT. Accepted candidates trigger a pruning step that removes highly dominated solutions (rank $\geq R_{\text{cutoff}}$) and enforces a hard archive size cap, followed by a full re-rank of the smaller remaining set. Rejected candidates are immediately removed and the rank array is restored from a snapshot, the *pop-on-reject* strategy described in the main text, preventing unbounded archive growth between accepted moves.

Algorithm S1 ParetoEnsembles: Pareto simulated annealing with incremental ranking

Require: User callbacks: Objective, Neighbor, Accept, Cool

Require: Initial parameter vector $\mathbf{x}_0$; hyperparameters N_{iter}, R_{cutoff}, $T_{\min}$, $n_{\max}$

Ensure: Archive of solutions with objective values, parameters, and Pareto ranks

$\mathbf{x}_{\text{best}} \leftarrow \mathbf{x}_0$
$\mathcal{A} \leftarrow \{(\mathbf{x}_0,\ \text{Objective}(\mathbf{x}_0))\}$; $\mathbf{R} \leftarrow [0]$; $T \leftarrow 1$

while $T > T_{\min}$ **do**
    for $t = 1, \ldots, N_{\text{iter}}$ **do**
        $\mathbf{x}' \leftarrow \text{Neighbor}(\mathbf{x}_{\text{best}})$; $\mathbf{f}' \leftarrow \text{Objective}(\mathbf{x}')$
        $\mathbf{R}_{\text{saved}} \leftarrow \text{copy}(\mathbf{R})$
        Append $(\mathbf{x}', \mathbf{f}')$ to $\mathcal{A}$; RankInsert$(\mathcal{A}, \mathbf{R})$
        if Accept$(\mathbf{R}, T) > U(0, 1)$ **then**
            Prune: $\mathcal{A} \leftarrow \{(\mathbf{x}_i, \mathbf{f}_i) \in \mathcal{A} \mid R_i < R_{\text{cutoff}}\}$
            $\mathbf{R} \leftarrow \text{FullRank}(\mathcal{A})$
            if $|\mathcal{A}| > n_{\max}$ **then**
                Keep only the $n_{\max}$ solutions with lowest R_i
                $\mathbf{R} \leftarrow \text{FullRank}(\mathcal{A})$
            end if
            $\mathbf{x}_{\text{best}} \leftarrow \mathbf{x}'$
        else
            Remove $(\mathbf{x}', \mathbf{f}')$ from $\mathcal{A}$; $\mathbf{R} \leftarrow \mathbf{R}_{\text{saved}}$
        end if
    end for
    $T \leftarrow \text{Cool}(T)$
end while
$\mathbf{R} \leftarrow \text{FullRank}(\mathcal{A})$
return $\mathcal{A}$, $\mathbf{R}$

Strict dominance test. The pairwise dominance check (Algorithm S2) determines whether one solution strictly dominates another by iterating over all m objectives. The test returns TRUE only if solution j is no worse than i on every objective and strictly better on at least one, which is the strict Pareto dominance relation that corrects the weak dominance used in the original POETs implementation. An early-exit condition terminates the loop as soon as j is found to be worse than i on any single objective, avoiding unnecessary comparisons.

Algorithm S2 StrictlyDominates($\mathbf{f}_j, \mathbf{f}_i, m$): Does solution j strictly dominate solution i?

Require: Objective vectors $\mathbf{f}_j, \mathbf{f}_i \in \mathbb{R}^m$ (lower is better)
Ensure: TRUE if $\mathbf{f}_j \prec \mathbf{f}_i$, FALSE otherwise

strictly_better $\leftarrow$ FALSE
for $k = 1, \ldots, m$ **do**
  if $f_{j,k} > f_{i,k}$ **then**
    return FALSE ▷ j is worse in objective k
  else if $f_{j,k} < f_{i,k}$ **then**
    strictly_better $\leftarrow$ TRUE ▷ j better in ≥ 1 objective
  end if
end for
return *strictly_better*

Incremental rank update. When a single candidate is appended to the archive, recomputing all pairwise dominance relationships would cost $O(n^2 m)$. The RankInsert procedure (Algorithm S3) exploits the fact that only one solution changed: it performs a single pass over the existing archive, testing dominance in both directions between the new solution and each incumbent. Existing ranks are incremented where the new solution dominates an incumbent, and the new solution's rank is accumulated from the number of incumbents that dominate it. This reduces the per-iteration cost to $O(nm)$, with a full $O(n'^2 m)$ re-rank deferred to the pruning step where the archive is smaller.

Algorithm S3 RankInsert($\mathcal{A}, \mathbf{R}$): Incremental rank update after appending one solution

Require: Archive $\mathcal{A} = \{(\mathbf{x}_1, \mathbf{f}_1), \ldots, (\mathbf{x}_n, \mathbf{f}_n)\}$ where $(\mathbf{x}_n, \mathbf{f}_n)$ is new
Require: Rank array $\mathbf{R} = [R_1, \ldots, R_{n-1}]$ valid for solutions $1, \ldots, n-1$
Ensure: Updated $\mathbf{R} = [R_1, \ldots, R_n]$ valid for the full archive

$R_{\text{new}} \leftarrow 0$
for $i = 1, \ldots, n-1$ **do**
  if StrictlyDominates($\mathbf{f}_n, \mathbf{f}_i, m$) **then**
    $R_i \leftarrow R_i + 1$ ▷ new solution dominates i
  end if
  if StrictlyDominates($\mathbf{f}_i, \mathbf{f}_n, m$) **then**
    $R_{\text{new}} \leftarrow R_{\text{new}} + 1$ ▷ i dominates new solution
  end if
end for
Append R_{new} to $\mathbf{R}$

Multi-chain parallel execution. Because simulated annealing is inherently sequential within a single chain, the most effective parallelization strategy is to run C independent chains from different starting points and merge the results (Algorithm S4). Each chain receives its own deterministic random number generator seeded from a base seed and the chain index, ensuring full reproducibility. After all chains complete, their archives are concatenated

and a final full ranking is performed on the merged set, producing a combined ensemble with broader coverage of the trade-off surface than any individual chain can achieve.

Algorithm S4 ParetoEnsemblesParallel: Multi-chain parallel execution

Require: Initial states $\{\mathbf{x}_0^{(1)}, \ldots, \mathbf{x}_0^{(C)}\}$; base seed s
Ensure: Merged archive with Pareto ranks

```
for c = 1, ..., C in parallel do
    rng_c ← Seed(s, c)
    (A_c, R_c) ← ParetoEnsembles(x_0^(c), rng_c)
end for
A ← A_1 ∪ ··· ∪ A_C
R ← FullRank(A)
return A, R
```

S2 Software design

ParetoEnsembles.jl is a pure Julia package with no dependencies beyond the standard library module `Random`, and its public API consists of five functions (Table S1). The primary entry point, `estimate_ensemble`, runs a single Pareto SA chain and returns the retained objective values, parameter vectors, and Pareto ranks; its parallel counterpart, `estimate_ensemble_parallel`, runs C independent chains concurrently, merges their archives, and re-ranks the combined set. A standalone `rank_function` computes Pareto ranks for an arbitrary $m \times n$ objective matrix and can be used independently of the optimization loop, while `hypervolume` computes the hypervolume indicator for a 2D front using a sweep-line algorithm in $O(n \log n)$ time and `pareto_front` extracts the rank-zero subset from the archive for downstream analysis.

The algorithm is defined by four user-supplied callback functions (Table S2): an objective function that maps a parameter vector $\mathbf{x} \in \mathbb{R}^d$ to an $m \times 1$ array of objective values, a neighbor function that perturbs a given parameter vector to generate a candidate, an acceptance probability function that returns a scalar probability from the rank array and temperature, and a cooling function that maps the current temperature to a reduced value. Beyond these four callbacks, the behavior of `estimate_ensemble` is controlled by keyword arguments including the pruning threshold `rank_cutoff` (default 5), the number of candidates per temperature level `maximum_number_of_iterations` (default 20), the stopping temperature `temperature_min` (default 10^{-4}), and the hard archive size cap `maximum_archive_size` (default 1000). When `trace=true`, the algorithm records convergence diagnostics (temperature, archive size, and hypervolume) at each cooling step, returning a fourth element in the output tuple that allows users to monitor convergence and assess whether the annealing schedule was sufficient.

Table S1: Public API of ParetoEnsembles.jl.

**Function**	**Description**
`estimate_ensemble`	Run a single Pareto SA chain. Returns objective values, parameter vectors, and Pareto ranks for the retained archive.
`estimate_ensemble_parallel`	Run C independent chains in parallel (one per starting point), merge archives, and re-rank.
`rank_function`	Compute Pareto ranks for an $m \times n$ objective matrix.
`hypervolume`	Compute the hypervolume indicator for a 2D Pareto front given a reference point.
`pareto_front`	Extract Pareto-optimal (rank = 0) solutions from the archive.

Table S2: User-supplied callback functions.

Callback	Signature	Purpose
`objective_function`	$(\mathbf{x}) \to \mathbb{R}^{m\times 1}$	Evaluate m objectives at parameter vector $\mathbf{x}$
`neighbor_function`	$(\mathbf{x}) \to \mathbb{R}^{d}$	Generate a candidate by perturbing $\mathbf{x}$
`acceptance_probability_function`	$(\mathbf{R}, T) \to [0,1]$	Acceptance probability given ranks and temperature
`cooling_function`	$(T) \to T'$	Annealing schedule

S3 Parallel execution

Two opportunities for parallelism arise from the structure of the algorithm. First, the outer loop of both the full ranking and the incremental RankInsert procedure iterates independently over archive solutions, so setting `parallel_evaluation=true` dispatches to threaded variants that distribute these independent iterations across available threads using Julia's `Threads.@threads` construct, with an atomic accumulator used for R_{new} in the incremental case since multiple threads contribute to it. Second, and more significantly, because simulated annealing is inherently sequential within a single chain, the most effective parallelization strategy is to run C independent chains from different starting points and merge the resulting archives (Algorithm S4); each chain receives its own deterministic random number generator seeded from a base seed and the chain index, ensuring reproducibility, and after all chains complete, the archives are concatenated and a final full ranking is performed on the merged set.

To quantify the benefit of multi-chain parallelism, we measured wall clock time for the cell-free ensemble estimation (ten chains, $N_{\text{iter}} = 50$) as a function of the number of Julia threads (Table S3). Each configuration was run five times and the median time is reported; the speedup is approximately linear up to four threads (3.1×) and reaches 4.1× at eight threads, with diminishing returns due to load imbalance when ten chains are distributed across eight threads and the serial cost of the final archive merge and re-ranking step.

Table S3: Multi-chain parallel scaling on the cell-free gene expression model (ten chains, $N_{\text{iter}} = 50$, median of five runs).

Threads	Wall clock (s)	Speedup
1	18.52	1.0×
2	9.38	2.0×
4	5.94	3.1×
8	4.51	4.1×

S4 Standard benchmarks

We validated ParetoEnsembles.jl on two standard multiobjective benchmarks: the constrained Binh–Korn problem [18] (Equation (3)) and the unconstrained Fonseca–Fleming problem [19] with $d = 3$ decision variables (Equation (4) and Figure S1). Constraints in the Binh–Korn problem are enforced via quadratic penalty terms added to the objective values, and bound constraints are handled by clamping in the neighbor function. We ran both benchmarks using ten parallel chains with $N_{\text{iter}} = 60$ candidates per temperature, a rank cutoff of $R_{\text{cutoff}} = 12$, and a cooling rate of $\alpha = 0.95$, and the resulting Pareto fronts in objective space recover the characteristic trade-off curves for both problems, with a visible cloud of near-optimal solutions surrounding the non-dominated front (Figure S1a,b). The computed front for the Fonseca–Fleming case lies directly on the theoretical curve, confirming that the algorithm converges to the correct solution set, and in parameter space the Binh–Korn Pareto-optimal decision vectors trace a curved manifold from the origin toward $(5, 3)$ while the Fonseca–Fleming solutions cluster along the diagonal $x_1 \approx x_2$ as expected from the symmetric structure of the objectives (Figure S1c,d). Comparing a single chain against ten parallel chains on the Binh–Korn problem illustrates the benefit of multi-chain execution (Figure S2): the single chain recovers the trade-off curve but, because every retained solution is non-dominated within that chain's archive, the rank array contains only rank-zero entries, whereas the merged multi-chain run produces a denser front with broader coverage of the feasible region.

$$\begin{aligned} \min_{\mathbf{x}} \quad & f_1 = 4x_1^2 + 4x_2^2, \qquad f_2 = (x_1 - 5)^2 + (x_2 - 5)^2 \\ \text{s.t.} \quad & (x_1 - 5)^2 + x_2^2 \leq 25, \qquad (x_1 - 8)^2 + (x_2 - 3)^2 \geq 7.7 \\ & 0 \leq x_1 \leq 5, \quad 0 \leq x_2 \leq 3. \end{aligned} \tag{3}$$

$$f_1(\mathbf{x}) = 1 - \exp\left(-\sum_{i=1}^{d}\left(x_i - \frac{1}{\sqrt{d}}\right)^2\right), \qquad f_2(\mathbf{x}) = 1 - \exp\left(-\sum_{i=1}^{d}\left(x_i + \frac{1}{\sqrt{d}}\right)^2\right). \tag{4}$$

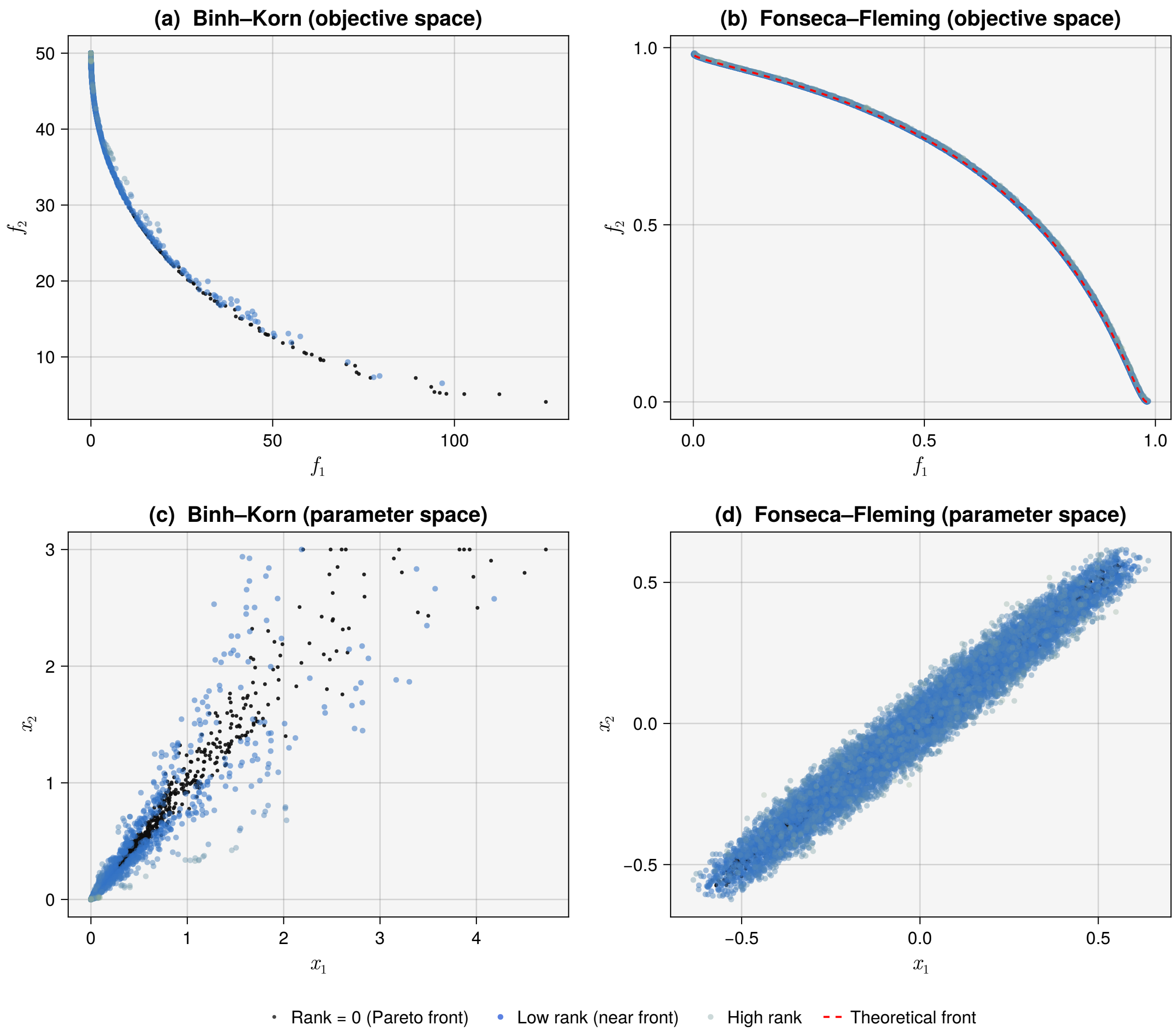

Figure S1: Benchmark results for (a,c) Binh–Korn and (b,d) Fonseca–Fleming ($d = 3$). Top row: objective space; bottom row: parameter-space projections (x_1 vs. x_2). Non-dominated solutions (rank $= 0$, dark) define the Pareto front, while near-optimal solutions (blue, colored by rank) form a cloud around it representing the retained ensemble. The dashed red curve in (b) is the theoretical Pareto front. Ten parallel chains were used with $R_{\text{cutoff}} = 12$, $N_{\text{iter}} = 60$, and $\alpha = 0.95$.

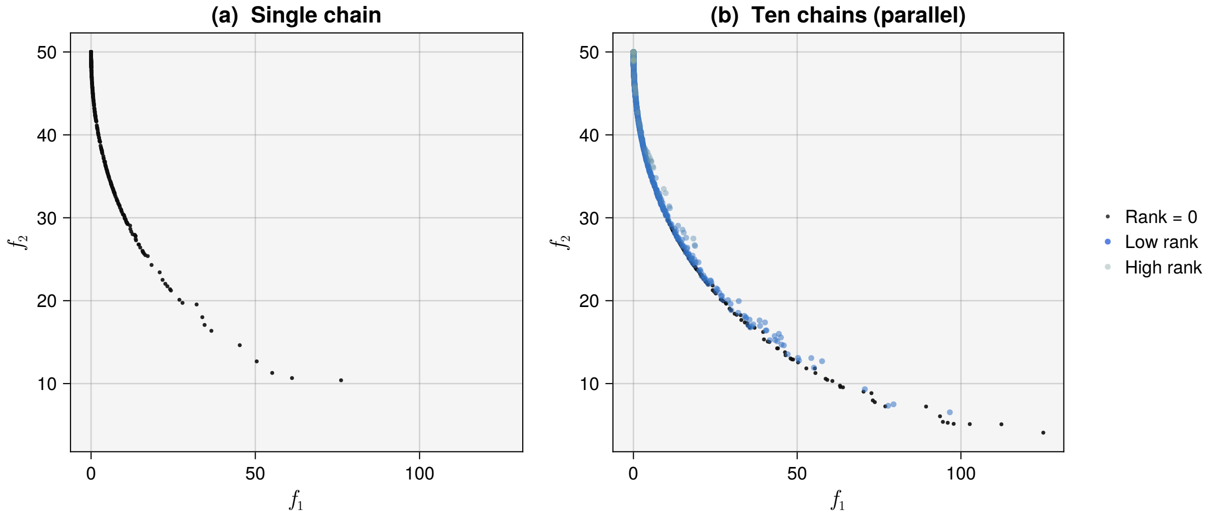

Figure S2: Comparison of (a) single-chain and (b) ten-chain parallel execution on the Binh–Korn problem. Both panels share the same axis limits. The single chain yields a sparse archive of non-dominated solutions, while the merged multi-chain archive provides denser front coverage and a visible cloud of near-optimal solutions (blue) surrounding the front.

S5 Comparison with NSGA-II

To position ParetoEnsembles.jl relative to population-based evolutionary methods, we compared it against NSGA-II [5] as implemented in Metaheuristics.jl [14] on both benchmark problems, matching the function evaluation budget at approximately 110,000 evaluations per solver (ParetoEnsembles: 10 chains, $N_{\text{iter}} = 60$ candidates per temperature, cooling rate $\alpha = 0.95$; NSGA-II: population 200, 550 generations). Five independent replicates were run for each solver, and the median hypervolume indicator (HV) and inverted generational distance (IGD) are reported in Table S4, with IGD computed against the analytical Pareto front for the Fonseca–Fleming problem and against a densely sampled reference front from a high-budget run for Binh–Korn. ParetoEnsembles.jl achieved comparable or higher hypervolume than NSGA-II on both problems and substantially lower IGD (5× lower on Binh–Korn and 7× lower on Fonseca–Fleming), indicating a closer approximation to the true Pareto front (Figure S3), while also producing much denser fronts with 2,000–6,000 non-dominated solutions compared with 200 for NSGA-II, reflecting its design as an ensemble generator that characterizes the full cloud of near-optimal solutions rather than merely approximating the front boundary. NSGA-II was 6–150× faster in wall time depending on problem size, consistent with its population-parallel evaluation strategy, but this speed advantage comes at the cost of a sparser representation of the trade-off surface that may be insufficient for downstream uncertainty analysis.

Table S4: Comparison of ParetoEnsembles.jl and NSGA-II on benchmark problems (median over 5 replicates). Higher HV and lower IGD are better. Both solvers used ∼110,000 function evaluations.

**Problem**	**Solver**	**HV**	**IGD**	**Front size**	**Time (s)**
Binh–Korn	ParetoEnsembles	**8035**	**0.014**	6172	77
	NSGA-II	8027	0.071	200	0.5
Fonseca–Fleming	ParetoEnsembles	**0.44**	**0.0004**	2051	3.1
	NSGA-II	0.44	0.003	200	0.5

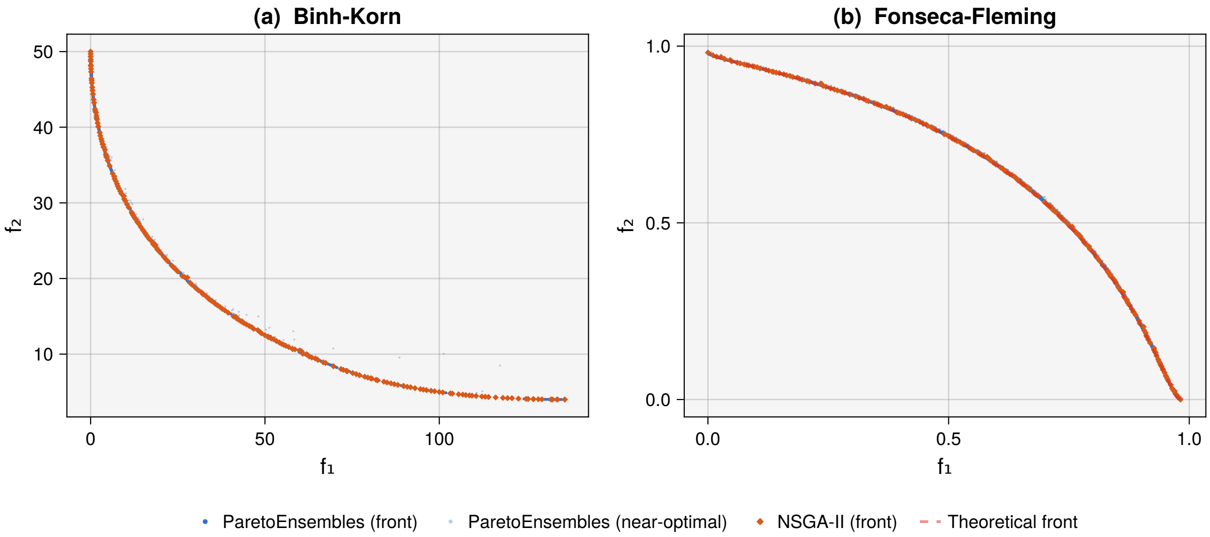

Figure S3: Comparison of ParetoEnsembles.jl (left) and NSGA-II (right) on (a,b) Binh–Korn and (c,d) Fonseca–Fleming at matched evaluation budgets (∼110,000). ParetoEnsembles.jl retains a cloud of near-optimal solutions (blue) in addition to the Pareto front (black), while NSGA-II returns only the non-dominated front. The dashed red curve in (c,d) is the theoretical Fonseca–Fleming front.

S6 Hyperparameter sensitivity

We assessed the sensitivity of ParetoEnsembles.jl to three key hyperparameters (rank cutoff R_{cutoff}, cooling rate α, and iterations per temperature N_{iter}) by sweeping each while holding the others at default values ($R_{\text{cutoff}} = 8$, $\alpha = 0.90$, $N_{\text{iter}} = 40$) on the Binh–Korn benchmark with five chains and five replicates per configuration (Figure S4). The hypervolume indicator was robust across all tested ranges: rank cutoff values from 2 to 12 produced nearly identical results, with a decline at $R_{\text{cutoff}} = 20$ where the archive retains many low-quality solutions; faster cooling ($\alpha = 0.80$) performed comparably to slower cooling ($\alpha = 0.95$), with the latter showing more variability; and even 10 iterations per temperature level produced competitive fronts, though 20 or more reduced inter-replicate variance. These results suggest that the default hyperparameters provide a reasonable starting point for most problems, and that users need not invest significant effort in tuning, a practical advantage for the systems biology applications where ParetoEnsembles.jl is primarily intended to be used.

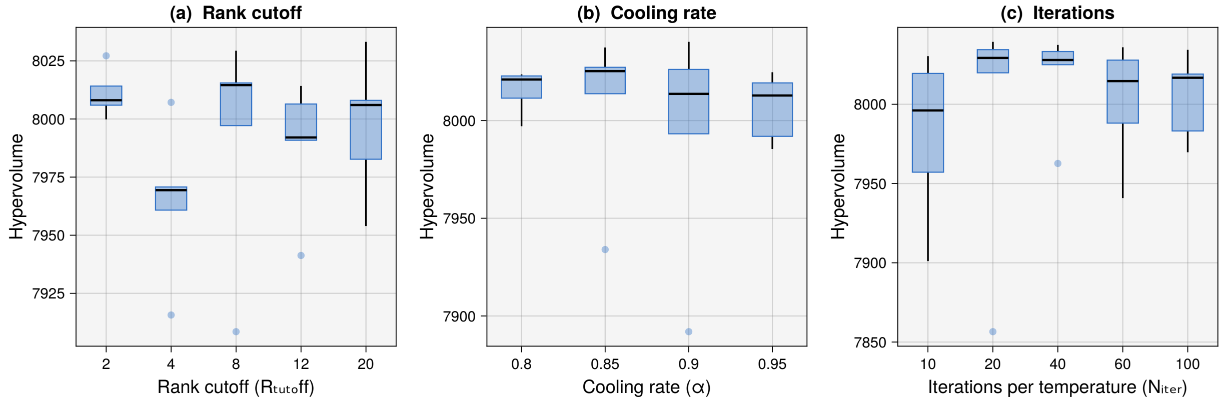

Figure S4: Hyperparameter sensitivity on the Binh–Korn benchmark. Box plots show the distribution of hypervolume over 5 replicates (median, interquartile range, and whiskers). (a) Rank cutoff R_{cutoff}, (b) cooling rate α, (c) iterations per temperature N_{iter}. Default values for the other two parameters are held constant in each sweep.

S7 Robustness to model misspecification

The coagulation results in the main text use the same model to generate and fit the data, so the fitting model is exactly correct by construction, a setting sometimes called an "inverse crime" in the inverse problems literature (Figure S5). To assess how the ensemble approach performs when this assumption is violated, we repeated the coagulation study with a deliberately misspecified model: six rate constants that are *not* among the ten estimated parameters were perturbed by ±30% from their nominal values before generating the synthetic training data, so that no parameter vector in the fitting model's search space can perfectly reproduce the data. The perturbed parameters span the initiation (TF=VIIa+VII→VIIa), binding (extrinsic Xase and IX activation K_m), assembly (prothrombinase on-rate), and inhibition (Xa+TFPI and mIIa+ATIII) pathways, ensuring that the misspecification affects multiple phases of the coagulation cascade.

Despite the model error, the ensemble still produced thrombin trajectories that closely tracked the training data at all three TF concentrations (Figure S5a), demonstrating that the ten estimated parameters absorbed much of the misspecification through compensatory adjustments. At the held-out validation conditions (10, 20, and 30 pM TF), the ensemble predicted peak thrombin to within 4–6% of the true values (Figure S5b), comparable to the 6–7% error observed with the correct model in the main text. However, trajectory-level coverage degraded substantially: the 95% prediction intervals covered the true trajectory at only 31–48% of time points, compared with higher coverage under the correct model, reflecting the ensemble's inability to account for structural model error through parametric uncertainty alone.

The pattern of TGA feature accuracy also shifted under misspecification (Figure S5c). Lag time, which was well-covered in the correct-model study, showed a systematic overprediction of 5–9% with the true value falling outside the 95% interval at some conditions, while ETP, which was biased under the correct model, was now predicted to within 0.3–2.5% with good coverage. Peak thrombin remained biased by 4–6%, similar to the correct-model case. These shifts reflect the fact that the estimated parameters compensate for the misspecified fixed parameters differently depending on which aspect of the thrombin curve is being evaluated. They also highlight a fundamental limitation of parametric uncertainty characterization: the ensemble can only explore parameter vectors within a given model structure, and when that structure is approximate, prediction intervals may be miscalibrated even though point predictions remain accurate. Parameter recovery (Figure S5d) showed wider scatter and larger biases than the correct-model case, as expected since the "true" parameter values are no longer optimal under the misspecified model.

These results confirm that the ensemble approach is robust to moderate model misspecification, with predictions degrading gracefully, while revealing the boundary between parametric uncertainty, which the ensemble captures, and structural uncertainty, which requires either model expansion or explicit model-error terms to address.

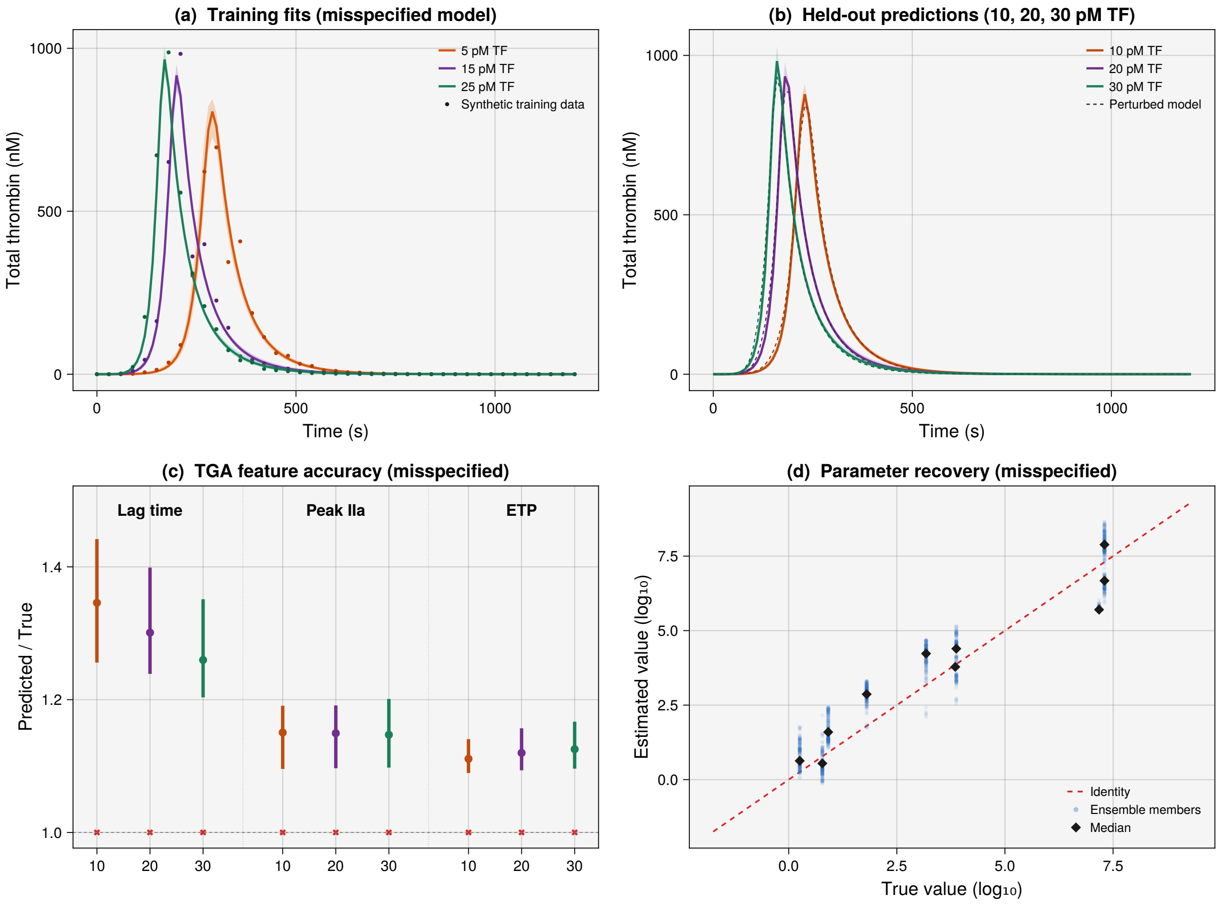

Figure S5: Model misspecification study: six fixed rate constants perturbed by ±30% before generating training data, then fitted with the nominal model. (a) Training fits at 5, 15, 25 pM TF; the misspecified model still tracks the data. (b) Held-out predictions at 10, 20, 30 pM TF; ensemble means (dashed) are within 4–6% of true trajectories (solid) but 95% CIs (shaded) do not fully bracket the truth. (c) TGA feature accuracy at held-out conditions, normalized to true values; the bias pattern shifts relative to the correct-model case. (d) Parameter recovery shows wider scatter and larger biases than the correct model, as expected when the fitting model is structurally approximate.

S8 Code examples

Listing 1: Binh–Korn benchmark with ParetoEnsembles.jl.

```
using ParetoEnsembles

function objective_function(x)
    f = zeros(2, 1)
    f[1] = 4.0 * x[1]^2 + 4.0 * x[2]^2
    f[2] = (x[1] - 5)^2 + (x[2] - 5)^2
    # penalty for constraint violations
    lambda = 100.0
    v1 = 25 - (x[1] - 5)^2 - x[2]^2
    v2 = (x[1] - 8)^2 + (x[2] - 3)^2 - 7.7
    f[1] += lambda * (min(0, v1))^2
    f[2] += lambda * (min(0, v2))^2
    return f
end

neighbor_function(x) = clamp.(
    x .* (1 .+ 0.05 * randn(length(x))),
    [0.0, 0.0], [5.0, 3.0])

acceptance_probability_function(R, T) =
    exp(-R[end] / T)

cooling_function(T) = 0.9 * T

(EC, PC, RA) = estimate_ensemble(
    objective_function, neighbor_function,
    acceptance_probability_function, cooling_function,
    [2.5, 1.5]; rank_cutoff=4.0,
    maximum_number_of_iterations=40, show_trace=false)

pareto_idx = findall(RA .== 0)
```

Listing 2: Multi-chain parallel execution on the Binh–Korn problem (requires `julia -t4`).

```
using ParetoEnsembles

# ... (same callbacks as Listing 1) ...

initial_states = [
    [2.5, 1.5], [0.5, 2.5],
    [4.0, 0.5], [1.0, 1.0]
]

(EC, PC, RA) = estimate_ensemble_parallel(
```

```
    objective_function, neighbor_function,
    acceptance_probability_function, cooling_function,
    initial_states;
    rank_cutoff=4.0, maximum_number_of_iterations=40,
    show_trace=false, rng_seed=42)

println("Solutions: ", size(EC, 2))
println("Pareto-optimal: ", count(RA .== 0))
```
